# First autochthonous dengue outbreak in Italy, August 2020

**DOI:** 10.2807/1560-7917.ES.2020.25.36.2001606

**Published:** 2020-09-10

**Authors:** Luca Lazzarini, Luisa Barzon, Felice Foglia, Vinicio Manfrin, Monia Pacenti, Giacomina Pavan, Mario Rassu, Gioia Capelli, Fabrizio Montarsi, Simone Martini, Francesca Zanella, Maria Teresa Padovan, Francesca Russo, Federico Gobbi

**Affiliations:** 1 Department of Infectious and Tropical Diseases, San Bortolo Hospital, Vicenza, Italy; 2 Veneto Region Arbovirosis Task Force, Venezia, Italy; 3 Department of Molecular Medicine, University of Padova, Italy; 4 Microbiology and Virology Unit, Padova University Hospital, Padova, Italy; 5 Department of Public Health AULSS8, Vicenza, Italy; 6 Department of Microbiology, San Bortolo Hospital, Vicenza, Italy; 7 Istituto Zooprofilattico Sperimentale delle Venezie, Legnaro, Padova, Italy; 8 Entostudio s.r.l., Ponte San Nicolò, Padova, Italy; 9 Direzione Prevenzione, Sicurezza Alimentare Veterinaria – Regione Veneto, Venice, Italy; 10 Department of Infectious/Tropical Diseases and Microbiology, IRCCS Sacro Cuore Don Calabria Hospital, Negrar, Verona, Italy

**Keywords:** Aedes albopictus, dengue, autochthonous, outbreak, surveillance, fever, arbovirosis

## Abstract

In August 2020, during the coronavirus disease (COVID-19) pandemic, five locally acquired cases of dengue virus type 1 were detected in a family cluster in Vicenza Province, North-East Italy where *Aedes albopictus* mosquitoes are endemic. The primary case was an importation from West Sumatra, Indonesia. This is the first outbreak of autochthonous dengue reported in Italy. During the COVID-19 pandemic, screening of febrile travelers from endemic countries is crucial in areas where competent vectors are present.

In Europe, dengue is almost always imported from endemic countries, and only very few autochthonous cases and limited outbreaks have been reported. We present the first autochthonous outbreak of dengue in Italy, due to an imported case from Indonesia.

## Case description

A woman in her thirties from Vicenza Province, Veneto region, Italy, (Case 1) stayed in Pulau Weh, a tropical island in West Sumatra, Indonesia, for 16 months and returned to her home town in Vicenza Province on 27 July 2020. She had a four-day stopover in Djakarta before flying back to Italy. After her return, she stayed at home for 14 days to complete the coronavirus disease (COVID-19) quarantine, mandated by the Italian government for travellers outside European Union (EU) countries at the time. Since 30 July, she experienced fever (38° C), malaise, back pain and upper limb itching. On 31 July, she was tested with oral and nasal swab for severe acute respiratory syndrome coronavirus 2 (SARS-CoV-2) RNA and found negative. Clinical symptoms resolved within 4 days.

Between 16 and 18 August, five of her seven household contacts started having symptoms of fever (> 38° C), malaise, headache, and upper limb itching. The contacts included a female and male in their fifties (Case 2 and Case 3, respectively), two males in their twenties, and a preschool child (Cases 4, 5 and 6, respectively). Clinical symptoms resolved within 4 days in all of them. Case 3 had stayed in in Pulau Weh in January 2020 for 14 days. She was asymptomatic during and after travel. Cases 2, 4, 5 and 6 never travelled abroad. Case 3 presented to the infectious diseases unit of our hospital on 21 August, when clinical symptoms had already disappeared. She was investigated for West Nile virus, Usutu virus, dengue virus (DENV), chikungunya virus (CHIKV) and Zika virus (ZIKV) by molecular and serology testing, because she reported that a family member (Case 1) had had similar symptoms after a recent travel in Indonesia. She was PCR tested for SARS-CoV-2 and found negative. The results were available on 22 August, and she was consecutively diagnosed with DENV type 1 (DENV-1) infection based on positive real-time reverse transcriptase (RT)-PCR on plasma, urine, and saliva, and positive DENV NS1 antigen in plasma, while serology testing was negative.

Following the dengue diagnosis of Case 3, Cases1, 2, 4, 5 and 6 were invited to present to the same unit, where they were tested on 26 August and also found positive for DENV-1 (Table). All serological tests for CHIKV, ZIKV, West Nile and Usutu Vires were negative. All patients recovered fully without complications, and none may be classified as severe dengue.

**Table:** Clinical and laboratory findings in outbreak (family cluster) of autochthonous dengue, Vicenza Province, Italy, July to August 2020 (n=6)

Clinical, epidemiological and laboratory parameters	Case 1	Case 2	Case 3	Case 4	Case 5	Case 6
Date of symptom onset	30 Jul	16 Aug	16 Aug	16 Aug	18 Aug	18 Aug
Delay between sample collection and onset of symptoms (days)	27	10	6	10	8	8
Symptoms	Fever (38° C), arthralgia, myalgia, headache	Fever (39° C), arthralgia, myalgia, headache	Fever (38° C), arthralgia, upper limb itching	Fever (38° C)	Fever (38.5° C)	Fever (39° C)
Epidemiological link	Source case	Household contact of Case 1	Index case and household contact of Case 1	Household contact of Case 1	Household contact of Case 1	Household contact of Case 1
DENV RNA in blood^a^	Negative	DENV-1	DENV-1	Negative	DENV-1	DENV-1
DENV RNA in urine^a^	Negative	DENV-1	DENV-1	DENV-1	DENV-1	DENV-1
DENV RNA in saliva^a^	Negative	Negative	DENV-1	Negative	DENV-1	DENV-1
DENV NS1 antigen^b^	Negative	Positive	Positive	Negative	Positive	Positive
DENV IgM^c^	Positive	Positive	Negative	Positive	Positive	Positive
DENV IgG^c^	Positive	Negative	Negative	Negative	Negative	Negative

RT: reverse transcriptase; US: United States..

^a^ For DENV RNA testing, total nucleic acids were purified from 200 μL of plasma, urine and saliva by using a MagNA Pure 96 System (Roche Applied Sciences, Basel, Switzerland). DENV RNA was amplified by in-house, real-time RT-PCR methods, which allowed discrimination between the different serotypes, according to the Center for Disease Control and Prevention protocol [13]. Real-time RT-PCR assays were carried out using the one-step, real-time kit (Thermo Fisher Scientific, Waltham, Massachusetts, US) and run on ABI 7900HT Sequence Detection Systems (Thermo Fisher Scientific).

^b^ DENV NS1 antigen was detected in plasma by using a rapid immuno-chromatographic assay (dengue NS1 Ag Strips, Bio-Rad, Hercules, California, US).

^c^ DENV IgM and IgG antibodies were detected by a chemiluminescence immunoassay (VirClia, Vircell, Granada, Spain) in a Thunderbolt instrument (Vircell).

## Vector control activities and public health measures

Following the notification to the public health authority of Veneto region on 26 August, public health technicians inspected the house where the affected family lives, which is in a rural, underpopulated area. On the same day, and for three consecutive days, mosquito control was conducted in the area comprised in a radius of 200m around the house, and extended to sensible places nearby such as a hospital and a touristic castle, as indicated by the National Plan against arbovirus infections [1].

Disinfestations included larvicides, adulticides and removal of the breeding sites. On 31 August, an entomological monitoring was set up using BG-sentinel traps, ovitraps and manual aspiration in order to control the effectiveness of the disinfestations, to define the mosquito density outside the area and to search for the virus.

A retrospective laboratory investigation was performed in all the 19 patients from Vicenza Province who were referred for suspected arbovirus infection during the previous month, which excluded DENV-1 infection in all of them.

General practitioners in the district where the outbreak occurred were alerted to refer cases of unexplained fever for investigation to the local infectious diseases department. During and soon after disinfestation activities, local health professionals, entomologists and representatives of the local police went from door-to-door in the radius of 200m to interview the inhabitants on symptoms, and raise awareness for the need to avoid mosquito bites and the need to remove possible larval breeding sites. The population of the city where the family resides is about 23,000 inhabitants, and was provided with informative brochures about dengue. In addition, on 28 August, the National Transplant Centre activated DENV NAT screening in organ, tissue and haematopoietic stem cell donors, as well as in blood donors, who reside in Vicenza Province or had stayed for at least one night in Vicenza Province in the 28 days before donation. Blood collections from donors in the town where the outbreak occurred have been suspended.

## Ethical statement

This analysis was conducted as part of public health usual practice, and was not conducted for research. Patients gave informed consent to anonymously publish their clinical and laboratory data for the purpose of this paper.

## Discussion

Between 2010 and 2019, autochthonous cases of dengue fever in Europe were reported in 2010 from France [2] and Croatia [3], in 2012 from Madeira, Portugal [4], from France in 2013, 2014, 2015, 2018, 2019 [5-7] and from Spain in 2018 and 2019 [6,7]. These cases were caused by DENV-1, as in cases described here, and DENV-2 [6].

Previously, in Italy no autochthonous cases of dengue had been reported, while two outbreaks of CHIKV disease were detected in Emilia-Romagna region and in central-southern Italy in 2007 and in 2017, respectively [8,9]. The Veneto region started an integrated surveillance for imported fevers in 2010 [10]. The surveillance, active between June and November, aims to increase the detection rate of DENV and CHIKV (since 2015, also ZIKV) infection in travellers from endemic areas and to promptly identify potential autochthonous cases. Patients with fever > 38° C in the last 7 days and recent (less than 15 days) return from endemic countries, after ruling out malaria, are referred to an infectious diseases department of the region to perform a rapid test for DENV and to collect samples for the regional reference laboratory in Padua, for second line testing. Entomologic investigations are also performed in the areas surrounding (200m) the residence of human cases of DENV, CHIKV and ZIKV infection, if notified as viraemic [11]. In ten years of surveillance (2010–2019), ca 1,400 patients were tested and 171 were positive for DENV, 35 for CHIKV and 21 for ZIKV. Each year, a variable proportion of febrile cases returning from endemic areas, ranging from 4.7% in 2016 to 24.5% in 2019, are diagnosed with an arbovirus infection. All these cases were imported.

A mathematical model was applied to epidemiological and entomological data to assess the risk of a DENV outbreak in northern Italy [12]. The risk for DENV outbreaks was estimated lower than for CHIKV, because of the lower competence for transmitting DENV of *Ae*. *albopictus*, the dominant vector in our area. We cannot exclude that unnoticed autochthonous cases of DENV may have occurred in Italy, especially if asymptomatic. However, we believe that any cluster of unexplained summer fever should be detected by existing clinical surveillance plans.

The index patient in the outbreak described here was not promptly referred to an infectious disease department, but the mandatory COVID-19 restriction measures also limited the risk of DENV spread from the index case during the viraemic phase. At the same time, the ongoing COVID-19 pandemic caused a diagnostic delay, as the first clinical suspicion was COVID-19. However, the high density of mosquitoes in the area where the source case (Case 1) was living, as well as her permanent presence at home due to quarantine, led to a high attack rate among household contacts. Active clinical surveillance in the control area, aimed at diagnosing other possible secondary cases is still ongoing, as well as entomological surveillance, including installation of ovitraps, larval search and manual aspiration of adult mosquitos within and outside the control area. We suggest that risk of further local spread may be possible, depending on the number and movement of potential viraemic people outside the control area before clinical diagnosis and on the local mosquito density. Clinical surveillance of summer fever and surveillance of febrile travellers returning from endemic areas should not be limited to rule out SARS-CoV-2 infection, and should include arbovirus screening in all countries where competent vectors are present.
